# Positive Correlation Between General Public Knowledge and Attitudes Regarding COVID-19 Outbreak 1 Month After First Cases Reported in Indonesia

**DOI:** 10.1007/s10900-020-00866-0

**Published:** 2020-06-24

**Authors:** Dina Keumala Sari, Rina Amelia, Ridha Dharmajaya, Liza Meutia Sari, Nadya Keumala Fitri

**Affiliations:** 1 Tropical Medicine Program Study, Faculty of Medicine, Universitas Sumatera Utara, Medan, Indonesia; 2 Public Health Department, Faculty of Medicine, Universitas Sumatera Utara, Medan, Indonesia; 3 Neurosurgery Department, Faculty of Medicine, Universitas Sumatera Utara, Medan, Indonesia; 4 Oral Medicine Department, Faculty of Dentistry, Universitas Syah Kuala, Banda Aceh, Indonesia; 5 Medical Study Program, Faculty of Medicine, Universitas Sumatera Utara, Medan, Indonesia

**Keywords:** Prevention, Transmission, COVID-19, Response, New cases, Public health

## Abstract

The increasing number cases of coronavirus disease (COVID-19) infections in the general population in Indonesia raises questions concerning the public’s knowledge and attitudes regarding this pandemic. To determine the correlation between the general public’s knowledge and attitudes regarding the COVID-19 outbreak 1 month after the first cases were reported in Indonesia. This cross-sectional study was conducted between early March and the end of April 2020 in the general population of Indonesia, beginning with the North Sumatra region, where the spread of COVID-19 in Indonesia began. Questionnaires were randomly distributed online in the red zone in Indonesia. Data were collected by collecting people’s responses to the questionnaire, which were distributed via WhatsApp (WA) application and were competed independently by the participants. A descriptive analysis was conducted to describe the demographic characteristics, knowledge, and attitudes of the general population. A total of 201 people had good knowledge (98%) and a positive attitude (96%) regarding the COVID-19 pandemic. The respondents had a negative attitude in relation to two aspects of the COVID-19 outbreak: having to always maintain a distance of 1.5 m when in crowds, and not being able to regularly exercise or eat nutritious food (78.6% and 79.1%, respectively). Most people in Indonesia have good knowledge and a positive attitude regarding the COVID-19 pandemic. However, negative attitudes were still found in this study, and as a result, transmission prevention measures cannot reach their maximum effectiveness by simply publicizing the increase in day-to-day cases to the general public.

## Introduction

At present, the world is facing a pandemic due to an increase in coronavirus disease (COVID-19) infections. Indonesia is now among the countries that have experienced attacks. China reported the occurrence of this new disease on 31 December, 2019, with the first case being discovered on 1 December, 2019. At the end of 2019, the office of the World Health Organization (WHO) in China received news that an unknown type of pneumonia was causing the disease. An acute respiratory infection that attacks the lungs was detected in Wuhan, Hubei, China [1–3].

The first casualty due to COVID-19 occurred on 11 January, 2020. China recorded a death in the population due to COVID-19, but approximately three weeks later, China also reported the first person who was able to recover from COVID-19. Cases of COVID-19 infection have spread throughout the world. Throughout the twentieth century, the world faced an abundance of cases of disease infection, which caused a number of epidemics, but only some of these caused pandemics. Indonesia is home to clusters of infectious diseases, ranging from diphtheria and influenza to malaria, and on 2 March, 2020, two people were confirmed as being infected with COVID-19 in the country [1, 4, 5].

The first case in Indonesia was recorded on 2 March, 2020. It was announced that two people aged 31 and 64 years were infected with COVID-19, and it was assumed that COVID-19 had entered the country in the 3rd week of January 2020. On 21 May, 2020, an increase of 973 new cases of COVID-19 was reported in Indonesia, for a total of 20,162 cases. Of these, 14,046 cases were treated (69.7%). In 4838 cases, the patients recovered (24.0%), and a total of 1278 reported cases of death (6.3%) were reported in 34 provinces and four major provinces, which were East Java, Jakarta, West Java, and North Sumatra, that this study was conducted. A graph of COVID-19 cases in Indonesia shows that they are increasing daily [5].

Various efforts to suppress the transmission rate of COVID-19 are being made. One such effort involves practicing social distancing in public to prevent any close contact, followed by increasing the hygiene of the public by asking people to regularly wash their hands, in the hope of destroying the lipid bilayer of the virus using soap, water, and 62–71% ethanol, which may decrease the virus’ infectivity [3, 6, 7]. Other actions, which are socialized through various media sources, such as television, news from the government, and other social media platforms, include asking people to stay at home, refrain from using public transportation, always wear masks, maintain a safe distance from people when in crowds, and refrain from going to areas with a high number of COVID-19 infections. People are constantly informed that they should exercise, eat nutritious foods, and change their lifestyles, for instance, from always being exposed to sunlight to staying at home, in attempt to improve their immunity [6, 8–10].

However, the knowledge provided is not in-line with the actions that should be taken by the general public. The consequences of this are economic hardship, which forces people to leave their house to work, and ignorance regarding the dangers of COVID-19 infection.

Therefore, in this research, we aimed to understand the general public’s knowledge and attitudes in relation to COVID-19, which may provide insights into the effectiveness of the precautionary steps associated with the outbreak. Human health behavior follows either a knowledge–attitude–behavior continuum model or a behavior–intention measure [1, 11, 12]. In either case, knowledge and attitudes are vital for human health when people take action for themselves. Thus, we explored the relationship between the general public’s knowledge and attitudes regarding the COVID-19 outbreak in Indonesia. The objective of this research was to illustrate the correlation between the general public’s knowledge and attitudes regarding the COVID-19 outbreak 1 month after the first cases were reported in Indonesia. We hope that with an understanding of this relationship, community understanding of the importance of preventing COVID-19 transmission will be emphasized as a preventative measure to maintain public health.

## Materials and Methods

This was cross-sectional study that was conducted from early March to the end of April 2020 by randomly distributing questionnaires in areas infected with the COVID-19 virus in Indonesia. These areas were divided into several different demographic areas: location 1, including Aceh, North Sumatra, West Sumatra, South Sumatra, the Riau Islands, and Jambi; location 2, including Jakarta, West Java, East Java, Middle Java, Banten, and Yogyakarta; and location 3, including Gorontalo, and Papua. The three areas with the highest infection rates in Indonesia were Jakarta (32.5%), East Java (13.0%), and West Java (9.8%).

The questionnaire was distributed via the social media application WhatsApp (WA) using a Google form. These questionnaires were randomly sent to all affected regions, and 245 participants filled it out. However, only 201 questionnaires were taken to be analyzed in accordance with the selection criterion. The aim of the selection criterion, namely, that the questionnaire be completed by a respondent aged 18–60 years, was to ensure that the respondents could understand the questions and answer the questions based on their knowledge and attitudes.

The questionnaire was compiled based on questions relating to people’s knowledge and attitudes regarding COVID-19 infection in Indonesia. The questionnaire was prepared in a language that was easily understood by the respondents, and the validity and reliability of the questionnaire were evaluated. The questionnaire consisted of three parts. The first section contained data on the demographic characteristics of the respondents, such as their age, location, sex, highest education level attained, occupation, and sources of information about COVID-19. The second part contained 10 questions about the respondents’ knowledge of COVID-19, with one correct answer per question. The third part contained 10 statements about the attitude of respondents regarding the prevention of COVID-19 infection, with possible answers including agree, doubt, or disagree. The respondents were assured that the information collected would remain anonymous. The respondents also agreed to fill out the questionnaire.

A validity test was conducted using product moment correlation techniques (significance < 0.05), and reliability was also tested using Cronbach’s α (Cronbach α = 0.75). For the data analysis, each correct answer was given a value of 1, while an incorrect answer was given a value of 0. Knowledge was considered to be good if the number of correct answers was more than 7; average if the number of correct answers was 4–6; and poor if the number of correct answers was less than 3. The attitude rating was considered positive if the number of correct answers was 6–10, whereas attitude was considered negative if the number of correct answers was 0–5. Continuous variables are expressed as continuous variables using the means ± SDs, and categorical variables are expressed as percentage proportions. To show the relationship between knowledge and attitudes, a Chi-squared test was used, and to show the correlation, a Spearman’s rho test was used. *p*-values < 0.05 were considered to be statistically significant. We used the SPSS program (version 11.5; SPSS Inc., Chicago, IL, USA) to perform the analysis. This study was carried out after ethical approval was obtained from the Health Research Ethics. The committee of Sumatera Utara University Medical School (No. 16/TGL/KEPK FK USU/2020) and all the participants agreed to the study procedures and expressed their willingness to fill in the Google form, which was sent via WhatsApp (WA).

## Results

This study included 201 eligible respondents who filled out the questionnaire completely and submitted it. The questionnaires were distributed using a Google form, with the expectation that the participants would spread the questionnaire to nearby friends. The exclusion criterion was participant under 18 or over 60 years old, and 44 respondents were excluded. This was because the respondents were either still underage, and we could not be certain that they understood the questions, or the respondents were too old, and it was considered that, at that age, the respondents would have difficulty in providing accurate answers.

### Characteristics of the Participants

Most of the study respondents were within the age range of 18–25 years (32.3%). The mean age of the respondents in this study was 35.59 ± 12.45 years, with the oldest respondent being 60 years old and the youngest being 18 years old. The study areas were scattered in several regions in Indonesia, and most of them are red zone areas, where COVID-19 cases have been found. The areas in which the people who filled out the questionnaires were living were those with the highest COVID infection rates (Table 1).

**Table 1:** Socio-demographic data on participants

Characteristics	Participants (*n* = 201) Percentage (%)
Age (years)	35.59 ± 12.45
Age classification	
18–25	65 (32.3)
26–35	40 (19.9)
36–45	45 (22.4)
46–55	38 (18.9)
56–60	13 (6.5)
Location area	
Aceh, North Sumatera, West Sumatera, South Sumatera, the Riau Islands, and Jambi	152 (75.62)
Jakarta, West Java, East Java, Middle Java, Banten, and Yogyakarta	45 (22.39)
Gorontalo, and Papua	4 (1.99)
Sex	
Male	93 (46.3)
Female	108 (53.7)
Highest education	
S3	3 (1.49)
S2	25 (12.48)
S1	116 (57.71)
Senior high school	55 (27.36)
Junior high school	1 (0.48)
Primary school	1 (0.48)
Occupation	
Employee	97 (48.26)
Non-employee	53 (26.37)
Student	37 (18.41)
Housewife	14 (6.96)
Sources of COVID-19 information	
Social media	102 (57.75)
Television news	159 (79.10)
Government banners	64 (31.84)
Neighborhood information	48 (23.88)

Most of the respondents were women (53.7%) and bachelor graduates (57.71%), and most of them had jobs as government or private employees (48.26%). As for the source of people’s information about COVID-19, television news (79.10%) was the most prevalent, and another notable source was social media (57.75%) (Table 1).

### Knowledge and Attitudes of Participants

The questionnaire contained questions about the respondents’ knowledge and aims, and was designed to evaluate whether the respondent had basic information about COVID-19 and its transmission. For the most part, the respondents knew about COVID-19, but there were still nine respondents who answered incorrectly (4.5%). Knowledge about the origin of COVID-19 infection was reported, and questions concerning the symptoms of COVID-19 infection were answered correctly by all the respondents (100%). Questions about the COVID-19 exposure period were answered incorrectly, with 21 respondents (10.4%) answering that it was within four hours or four weeks. Most respondents knew the cause of exposure to COVID-19 (93%), but there were still 1.5% who did not understand the precautions and 8% who did not know how to increase immunity. Most respondents knew the symptoms of COVID-19 and the conditions that aggravate COVID-19 infection, but not all of them knew about COVID-19 symptoms (Table 2).

**Table 2:** Knowledge of participants

No.	Question (one best answer)	Correct answer percentage (%)
1	Is there a current pandemic due to a virus?	192 (95.5)
2	COVID-19 was first discovered in Indonesia?	201 (100)
3	What is the name of the animal that is believed to be the cause of the spread of COVID-19?	201 (100)
4	What are the symptoms of people affected by COVID-19?	180 (89.6)
5	How long is the COVID-19 exposure period?	197 (98)
6	What are the main causes of someone being exposed to COVID-19?	187 (93)
7	How can COVID-19 virus be prevented in the body?	198 (98.5)
8	What is one of the actions to increase immunity?	185 (92)
9	What do you do if you experience symptoms similar to those of COVID-19?	182 (90.5)
10	What are some of the circumstances that can aggravate COVID-19 infection?	196 (97.5)
Knowledge regarding COVID-19 (mean ± SD)		9.55 ± 0.73
Good		197 (98)
Medium		4 (2)

The questionnaire also assessed the attitudes of the respondents regarding COVID transmission and what conditions can reduce COVID-19 transmission. Most respondents responded well to the questions given by agreeing or disagreeing. As for the percentage of answers, the respondents had the worst attitude toward maintaining a distance of 1.5 m from others when shopping, working, studying, or worshiping (78.6%). They also had a negative attitude toward doing sporting activities and eating nutritious food (79.1%). Both of these attitudes indicate that the respondents experienced great difficulty in changing their daily habits, for instance, in maintaining social distancing regulations and increasing immunity (Table 3).

**Table 3:** Attitudes of participants

No	Question (one best answer)	Correct answer percentage (%)
1	I engage in activities outside of the home during this COVID-19 plague (no)	177 (88.1)
2	In carrying out activities outside in public, I use public transportation (online vehicles, public transportation, buses, or trains) (no)	179 (89.1)
3	When gathering or interacting, I always use a mask (yes)	161 (80.1)
4	I shake hands with others when I meet as usual (no)	187 (93)
5	I clean my hands with a wet hand sanitizer/tissue before handling the steering wheel of a car/motorcycle (yes)	190 (94.5)
6	I keep a 1.5 m distance from others when shopping, working, studying, or worshipping (yes)	158 (78.6)
7	I am in a village area where a patient is infected (No)	173 (86.1)
8	I always provide wet tissues/antiseptic, masks, and antiseptic soap for my family at home (yes)	186 (92.5)
9	I always try to be exposed to sunlight for at least 15 min every day (yes)	171 (85.1)
10	I engage in sporting activities and eat nutritious food (yes)	159 (79.1)
Attitude regarding COVID-19 transmission (mean ± SD)		8.66 ± 1.47
Positive		193 (96)
Negative		8 (4)

### Correlation Between Knowledge and Attitudes

Based on the data analysis, 197 respondents were included in the category of good knowledge (98%), and 4 had average knowledge (2%). The attitudes were reported to be mostly positive, totaling 193 respondents (96%) and only 8 respondents had negative attitudes (4%). Chi-squared analysis did not show a relationship between knowledge and attitudes (*p* = 0.151).

However, after the data were collected, an analysis was conducted using the Spearman’s rho test. A relationship between knowledge and community attitudes towards COVID-19 was shown, with a significance value of 0.04 (*p* < 0.05). These results indicated that good knowledge was correlated with a positive attitude, with a correlation value of 0.148, which indicated that the correlation was very weak.

## Discussion

Until now, the number of COVID-19 infections has been increasing, but in some countries, the rate of infections has decreasing, and in others, there has been no new infections. The general public in Indonesia is vulnerable to the fast transmission of COVID-19 if they are not properly informed. Age is a concern, especially the elderly with co-morbidities, but not all elderly people understand and want to get information about preventing COVID-19 transmission.

The elderly group is more vulnerable, but this group does not have sufficient access to the available information. Conversely, because the young age group has access to information from various sources, they can easily afford to obtain correct information about COVID-19 [7, 13].

COVID-19 is distributed throughout all regions in Indonesia, including the three regions with the highest prevalence: DKI Jakarta, East Java, and West Java. The other regions experienced an increase in COVID-19 cases, but the rate was not as fast as it was in these three regions [5]. It is expected that the public can comply with the various information and regulations issued by the government, but even if they do so, the prevalence rate will continue to increase, and this means that several factors are influencing the knowledge and attitudes of the general public in Indonesia. In this study, we considered the problems acting as obstacles to the affirmation of attitudes that facilitate the prevention of COVID-19 transmission.

Based on this study, the majority of women paid more attention when filling out the questionnaire compared to men. This might also have affected changing attitudes toward preventing COVID transmission. In this study, we also showed that it is easier for employees to obtain information and change their attitudes. In previous studies, younger people were found to more easily change their attitudes [2, 7, 14].

The most widely used sources of information are television and social media, with the highest being television. At present, social media plays an important role as a source of information, but it appears that television is considered a source of true and trustworthy information by the general public in Indonesia. Other studies have shown that, conversely, social media ranks first as a source of knowledge [12, 14]. This is likely due to social media providing information more quickly, allowing for the free disclosure of information, and varying without broadcasting censorship. Its only drawback is that not all of the information disseminated is credible because it is publicized without any assessment of its truth.

The findings in this study are relevant for the evaluation of the knowledge of the general public. We found that the percentage of incorrect answers was the highest for questions pertaining to the actions that need to be taken when experiencing COVID-19 symptoms. Most respondents answered that an infected person should be taken to the authorities instead of to the hospital. Thus, from this error, we found that COVID-19 infection has led to a new perspective, i.e., that this very rapid spread requires decisive action, especially from the police. It is interesting that some people do not focus on the disease or symptoms that arise, but rather on how to prevent someone who has COVID-19 from transmitting it to others. Sometimes, someone who has tested positive for COVID-19 does not want to be hospitalized because they think that the disease is very contagious, but they often unconsciously transmit the virus. Things like this ultimately make the police intervene, and this also disturbs the general public.

There is another question relevant to increasing immunity. Among others, the right answer is sunbathing, but a wrong answer was often given, for example, consuming more carbohydrates and drinking cold water. Sunlight can activate vitamin D under the skin and is reported to increase body immunity. Some previous studies link vitamin D and infectious diseases, such as tuberculosis, and report that vitamin D is associated with an improvement in the clinical symptoms of the disease [15–17].

One of the questions that shows the general public’s ignorance is the main cause of exposure to COVID-19. A small portion of the respondents selected air and food. These two were possible answers to the question concerning COVID-19 transmission, but coughing or sneezing are more accurate answers. Previous studies reported that not all respondents gave correct answers about how this virus can spread [12, 14]. Our evaluation of the knowledge of the respondents showed that there are three main problems with the general public’s knowledge of COVID-19. These problems pertain to action when symptoms are found in cases of COVID-19 infection, ways to increase immunity, and the main cause of exposure to COVID-19 in a person.

Based on the respondents’ attitudes toward COVID-19 infection, we found that people have the worst attitude regarding keeping a distance of 1.5 m from others, exercising and eating nutritious food, and wearing a mask when gathering with others. This is likely because the respondents have not experienced the conditions of the COVID-19 pandemic. Social distancing actions and quarantine situations produce major changes in the social fabric of countries, and this does not change quickly. However, previous studies reported that people agreed with the avoidance of transmission and strongly agreed with the isolation process and the need for an available vaccine [12]. Previous studies also reported that not all people agreed with the measures to prevent COVID-19 transmission, such as washing their nose with saline, gargling, and taking antibiotics [14].

Maintaining a distance of 1.5 m from other people is difficult to implement according to respondents from the general public. Changing this attitude is difficult, especially for people engaged in many social activities, such as shopping centers, parties, or education centers. The need for this change, which requires time, caused some respondents to disagree with the distance limitation.

The attitude that people still do not approve of is the attitude of exercising and eating nutritious food. This attitude is not considered to be directly related to COVID-19 infection, so people often do not approve of it. The body’s immunity increases with proper nutrition. Previous studies mentioned several food sources that can provide immunomodulatory effects, and respondents agreed with the benefits of these herbal foods [18, 19].

Some respondents still did not approve of wearing a mask when gathering with others. The use of masks is important for preventing COVID-19 transmission, as the main source of transmission is coughing or sneezing. This might also be due to it being unusual to cover the mouth and nose with a mask or due to the unavailability of masks. This attitude needs to be scrutinized and used as a basis for establishing a COVID-19 transmission prevention policy.

Knowledge is an important factor for forming one’s attitudes, but this is not absolute because various factors influence a person’s knowledge and attitudes. Attitudes are also not the only factor pertaining to changes in one’s lifestyle because there are other actions that can cause lifestyle changes. An attitude is a reaction of a person to stimulus. It is a predisposition to action and a readiness to react to certain environmental objects. Based on previous research, a relationship exists between knowledge and attitudes in dealing with the COVID-19 pandemic [6, 12, 20, 21].

A correlation between knowledge and attitudes was found in this study. It is a very weak correlation, but it is hoped that it will lead to the uncovering of the cause of community disobedience in preventing COVID-19 transmission 1 month after the COVID-19 infection was reported in Indonesia. If this can be explored and developed in larger research studies, then the number of COVID-19 cases in Indonesia may be suppressed.

This study has a limitation: not all of the respondents in the red zone area were able to fill out a questionnaire, but we hope that the results presented in this study regarding the knowledge and attitudes of the general public can describe those of the actual population. The results of this study are expected to be further developed with more in-depth research on knowledge and attitudes, especially knowledge and attitudes that are still not understood by most of the general public.

## Conclusion

The results of this study indicate that the general public has good knowledge and a positive attitude toward the COVID-19 pandemic. However, the general public still does not have answers to some knowledge questions and some have negative attitudes regarding COVID-19. Thus, transmission prevention measures cannot reach their maximum effectiveness by simply publicizing increases in daily cases in the general public.
